# Learning Curve of First-Trimester Detailed Cardiovascular Ultrasound Screening by Moderately Experienced Obstetricians in 3509 Consecutive Unselected Pregnancies with Fetal Follow-Up

**DOI:** 10.3390/life14121632

**Published:** 2024-12-09

**Authors:** Tibor Elekes, Gyula Csermely, Krisztina Kádár, László Molnár, Gábor Keszthelyi, Andrea Hozsdora, Miklós Vizer, Marianna Török, Petra Merkely, Szabolcs Várbíró

**Affiliations:** 1 RMC-Fetal Medicine Centre, Gábor Áron Street 74-78, H-1026 Budapest, Hungary; drelekestibor@gmail.com (T.E.); drcsermelygyula@gmail.com (G.C.); krisztinakadar@hotmail.com (K.K.); molnar.sumi@gmail.com (L.M.); keszimed2000@t-online.hu (G.K.); hozs.andi@gmail.com (A.H.); 2 Cardiovascular Medicine and Research Division, Semmelweis University, Üllői Street 26, H-1085 Budapest, Hungary; 3 DaVinci Private Hospital, Málics Ottó Street 1, H-7635 Pécs, Hungary; vizermiklosgabor@gmail.com; 4 Department of Obstetrics and Gynecology, Semmelweis University, Üllői Street 78a, H-1082 Budapest, Hungary; merkely.petra@gmail.com (P.M.); varbiro.szabolcs@semmelweis.hu (S.V.); 5 Workgroup of Research Management, Doctoral School, Semmelweis University, Üllői Street 22, H-1085 Budapest, Hungary; 6 Department of Obstetrics and Gynecology, University of Szeged, Semmelweis Street 1, H-6725 Szeged, Hungary

**Keywords:** cardiovascular ultrasound screening, learning curve, moderately experienced obstetricians, fetal follow up

## Abstract

Our primary objective was to assess the effectiveness of detailed cardiovascular ultrasound screening during the first trimester, which was performed by obstetricians with intermediate experience. We collected first-trimester fetal cardiac screening data from an unselected pregnant population at RMC-Fetal Medicine Center during a study period spanning from 1 January 2010, to 31 January 2015, in order to analyze our learning curve. A pediatric cardiologist performed a follow-up assessment in cases where the examining obstetrician determined that the fetal cardiac screening results were abnormal or high-risk. Overall, 42 (0.88%) congenital heart abnormalities were discovered prenatally out of 4769 fetuses from 4602 pregnant women who had at least one first-trimester cardiac ultrasonography screening. In total, 89.2% of the major congenital heart abnormalities (27 of 28) in the following fetuses were discovered (or at least highly suspected) at the first-trimester screening and subsequent fetal echocardiography by the pediatric cardiology specialist. Of these, 96.4% were diagnosed prenatally. According to our results, the effectiveness of first-trimester fetal cardiovascular ultrasound screening conducted by moderately experienced obstetricians in an unselected (’routine’) pregnant population may reach as high as 90% in terms of major congenital heart defects, provided that equipment, quality assurance, and motivation are appropriate.

## 1. Introduction

Moderate and severe congenital heart defects (CHD) requiring intensive postnatal cardiac care are the most frequent fetal malformations with an incidence of 6–8 per 1000 live births. If we consider all small-sized ventricular septal defects (VSD), bicuspid aortic valves, and other minor cardiac malformations, which potentially increase cardiac morbidity at later ages, this incidence may be as high as 7.5 per cent [1]. Fetal cardiac anomalies are responsible for 20% of intrauterine fetal demise and 20 to 30% of neonatal mortality. Prenatal diagnosis of CHD may help the work of genetic counselors, contribute to a better understanding of the prognosis of affected fetuses, and reduce the psychological and physical risk of affected pregnant women [2,3,4]. In utero detection of CHD may improve neonatal morbidity and mortality as a result of prior organization of intensive postnatal cardiac care [5,6,7,8,9]. Fetal echocardiography in high-risk patients is usually performed by pediatric cardiologists. The high-risk pregnant population includes pregnant women with a positive family history of CHD, in vitro fertilization, maternal metabolic disorder, mainly diabetes and obesity, higher maternal age, autoimmune diseases (SLE, Sjögren), fetal teratogen exposure, and abnormal NT, TR, and DV on the first-trimester genetic ultrasound examination [10,11,12,13,14,15]. In this group of high-risk pregnant patients, the vast majority of severe CHD cases are diagnosed prenatally [16,17]. On the other hand, according to the literature, 90% of CHD is not present in the high-risk group and routine obstetric ultrasound screening of the low-risk pregnant population is generally ineffective in finding the majority of major fetal cardiac anomalies due to the time-consuming nature of the examination and the lack of the acquired expertise, which would be greatly improved after a proper learning process [18,19,20,21,22,23]. Aneuploidy screening in the 11 to 13 + 6-week window by Fetal Medicine Foundation (FMF)-certified obstetric sonographers resulted in improved detection rates of CHD in the first trimester [24].

Given the fact that it is the obstetric sonographer who initially evaluates the 11–13-week fetus, there is great demand for obstetricians to conduct early and comprehensive fetal heart scans [25,26,27].

Thanks to the widespread use of high-frequency ultrasound scanners and the introduction of novel Doppler techniques, obstetricians skilled in fetal cardiac scanning are able to visualize four-chamber views and ventricular outflow tracts of first-trimester fetuses in approximately 95 to 97% of cases [28,29,30,31]. A significant number of international investigational research groups have proved high effectiveness (>90% detection rate, DR) of prospective first-trimester extended fetal cardiac screening conducted by highly qualified obstetric sonographers in high-risk pregnant patients [28,32]. However, the current literature lacks sufficient information regarding the effectiveness of early fetal cardiac examinations performed by obstetricians with basic and intermediate experience in unselected (low- and high-risk), also referred to as ’routine’ pregnant populations, and of the learning curve [31,33,34].

Our study’s primary goal was to assess the efficacy of first-trimester prolonged cardiovascular ultrasonography screening carried out by obstetricians with a moderate level of experience (an examiner experienced in the differential diagnosis of regular first-trimester heart planes but less experienced in the differential diagnosis of irregular first-trimester heart planes (i.e., has seen few abnormal cases)) in consecutive cases of an unselected pregnant population. Our investigation’s second goal was to examine and better understand the learning curve we saw throughout the course of the five-year study period with reference to the sonographic assessment of the fetal heart between weeks 11 and 13.

## 2. Materials and Methods

Our retrospective study was approved by the Scientific and Investigational-Ethical Committee of the Hungarian Medical Scientific Council (2013/EKU (588/2013)). Data from the first-trimester fetal cardiovascular screening of consecutive patients from an unselected (or “routine”) pregnant population at the RMC-Fetal Medicine Center throughout a study period from 1 January 2010 to 31 January 2015, were used to analyze the learning curve. Fetuses of pregnant women who received screening between 1 January 2011 and 30 June 2014, were monitored throughout pregnancy in order to assess the effectiveness of first-trimester cardiac screening.

Fetal cardiac scans were performed during routine first-trimester extended ultrasound screening, which included a comprehensive study of fetal sono-anatomy and aneuploidy, as well as preeclampsia screening in accordance with the protocol of the FMF (maternal age, body weight and height, maternal serum beta-hCG and PAPP-A assay combined with fetal sonographic measurement of nuchal translucency (NT), nasal bone (NB) visualization, fetal heart rate (FHR) measurement, tricuspid regurgitation (TR), ductus venosus (DV) blood flow examination for aneuploidy, and maternal blood pressure, medical history, serum PAPP-A, and, as of 1 January 2014, serum PlGF assay combined with sonographic assessment of maternal left and right uterine artery Doppler-flow PI-value for preeclampsia screening, respectively) [33,35].

Transabdominal ultrasound examinations were conducted by using Accuvix V20 (Samsung-Medison, Seoul, Republic of Korea) and Voluson S8 (GE Medical Systems, Florecne, SC, USA) ultrasound scanners both equipped with 4 to 8 MHz convex abdominal transducers. At the beginning of the study period, ultrasound examinations were performed by three obstetricians (all of them were FMF-audited sonographers for NT, NB, DV, and TR), who were experienced in the assessment of normal fetal first- and second-trimester cardiac anatomy but only moderately experienced in the final evaluation of abnormal fetal cardiac anatomy. During the last year of the study period, an FMF-audited sonographer joined the obstetric team, who pre-screened pregnant patients.

Intrauterine cardiac screening was introduced in the 1980s, which at that time only consisted of the examination of 4 cavity planes, ideally at 18–22 weeks. Subsequently, it was recognized that in many cases, CHD exists despite the normal four-cavity view; therefore, by further increasing the detection rate, in 2001, Yagel et al., added the determination of the abdominal situs, aortic arch, ventricular outflow tract, and mediastinal vessel examination as part of the screening procedure. In 2015, the three-vessel tracheal view was incorporated into the UK screening protocol. During our screenings, we used all of the aforementioned techniques in the first-trimester heart examination [36,37,38,39].

In all cases, a comprehensive medical history was taken from the patients, including maternal body weight and height, temperature, and blood pressure. As of 1 January 2011, an institutional standardized protocol was introduced for sonographic examination of the first-trimester fetal heart, according to which the following planes, structures, and functions are to be examined and archived digitally:

1. Determination of abdominal situs;
2. Cardiac size and axis;
3. Four-chamber view: crux cordis, ventricular septum in the four-chamber-plane, ventricular color inflow, tricuspid pulsed-wave Doppler, and FHR;
4. Three-vessel view, V-confluence of color flow;
5. Longitudinal view: aortic arch with color flow, ductal arch, right ventricular outflow tract, imaging of the crossing of great vessels (during first-trimester cardiac screening it is more reliable to visualize a cross-section of the aorta next to the longitudinal section of the pulmonary trunk from a median-sagittal plane rather than using an axial cardiac grand-sweep);
6. Optional examinations of the left atrial pulmonary vein (at least two) and right atrial caval vein connections (veno-atrial concordancy).

Cardiac screening according to this protocol was considered comprehensive if all of the previously mentioned structures were stored digitally. No time limitations were applied during the examinations. The examination was only interrupted when a visualization obstacle other than fetal position occurred, such as a significant distance between fetus and transducer (retroflected uterus, thick maternal abdominal wall, previous abdominal scars, anterior placenta), and where vaginal ultrasound was also found to be inadequate [40,41]. In cases deemed to be within the normal range at first-trimester cardiac screening, if the patient returned to us later, the cardiac scan was repeated at 18 to 20 weeks and at 28 to 30 weeks. A pediatric cardiologist reevaluated all routinely screened cases that were found to be “abnormal”, as well as high-risk instances. The parents received thorough genetic counseling in the case that the prenatal cardiac screening revealed abnormalities.

Fetal karyotyping by chorionic villous sampling or amniocentesis was offered to all pregnant women who had positive first-trimester aneuploidy screening test results or if the type of CHD indicated it.

Pregnant women who underwent screening at our center received routine prenatal, genetic, and general obstetric care at various institutes of the country (221 attending physicians); therefore, data concerning the outcomes of pregnancies were collected from patients in three different methods:

1. Patients responded to our queries via email (our address appeared on the first-trimester obstetric ultrasound report);
2. Data were retrieved from the digital records of the obstetric division of our center where patients received prenatal care;
3. Verbal interviews with mothers by phone were performed by trained health personnel of our center. In the event of inconclusive outcome data, a follow-up interview via phone was repeated by the obstetrician to ascertain clear outcome results.

For statistical analysis, the Chi-Square Test was used and *p* < 0.05 was evaluated as a significant difference. The data were evaluated using GraphPad Prism 6.0. software.

## 3. Results

Overall, 42 (0.88%) cases of congenital heart disease were identified during the 4769 fetuses (155 twins, 6 triplets, and 4441 singletons) of 4602 pregnant mothers who underwent at least one first-trimester cardiac ultrasound screening between 1 January 2010, and 31 January 2015 (the majority also underwent second and third-trimester screening).

Table 1 presents the summary of the distribution of different types of CHD according to the timing of detection and gestational age.

Considering the low case number (n = 228) observed in the first study year (2010) and the absence of a uniformly applied examination protocol for the assessment of the fetal heart, fetuses screened during this particular time interval were not followed. In the group of patients who underwent screening in the second half of 2014 (n = 1032 fetuses), the vast majority of pregnancies were still ongoing at the time of the outcome data analysis (from the 1 February 2015 to 31 March 2015). Consequently, only fetuses screened between the 1 January 2011 and the 30 June 2014 were followed and data retrieved from the outcome of the aforementioned fetuses were used to assess the effectiveness of screening.

During this three-and-a-half-year period, 3509 fetuses (125 twins, 6 triplets, and 3241 singletons) of 3372 pregnant women underwent a comprehensive fetal cardiovascular ultrasound screening at 11–13 + 6-weeks of gestation. The ratio of followed pregnancies was 93% (3142/3372). Of the 239 fetuses from 230 pregnancies who were lost at follow-up, no abnormalities were identified by a first-trimester fetal cardiac ultrasound scan. Of the 3270 followed fetuses, 3020 were singletons, 116 were twins, and 6 were triplets. The mean maternal age at the time of first-trimester screening was 33.9 years (range: 17–45). The ratio of pregnant women aged > 35 years was 46.9%. The mean fetal crown-rump length (CRL) was 64.0 mm (range: 45.0–84.0).

The mean maternal ages at the time of first-trimester screening in the entire study population and in the group of pregnancies affected with major fetal CHD were 33.9 and 35.8 years, respectively (nonsignificant, χ^2^ = 3.601, df = 1, *p* = 0.058). The proportions of women above 35 years of age in the overall study population and in the group of pregnancies affected by major fetal CHD were 46.9% and 71.0%, respectively (significant, χ^2^ = 6.642, df = 1, *p* = 0.0099).

The results of our NT, DV, and TR measurements (FMF software version 2.3) performed in conjunction with fetal cardiac screening revealed that 56.1% of NT values were above the median and 4.9% of them were above the 95th percentile, respectively. Simultaneously, in 54% of prenatally detected cardiac defects (20/37), NT-values were below the 99th percentile (<3.5 mm), and in 46% of them (17/37), the NT-value was less than 2.5 mm. With respect to other cardiac markers, TR was found in 0.6% of non-CHD cases and in 29% of fetuses with CHD (10/35) (significant, χ^2^ = 308.486, df = 1, *p* < 0.05. Similarly, an abnormal DV blood flow pattern was identified in 4.3% of all examined fetuses and in 51% of fetuses affected with cardiac anomalies (18/35) (significant, χ^2^ = 168.282, df = 1, *p* < 0.05).

### 3.1. Prenatal Diagnosis

During the first-trimester cardiac ultrasound screening of 3270 followed fetuses, 34 hearts were identified by the examining moderately experienced obstetricians as ‘abnormal’. Of the cases, two were deemed to be within the normal range (#33 and 34), the initial diagnosis was either refined or supplemented in five(#4, 14, 21, 24, and 32), and three cases were not examined (#6, 15, and 18) by the pediatric cardiology specialist. In 24 cases, the cardiologist agreed with the initial diagnosis. In the three non-examined cases by pediatric cardiology specialists, pregnant women did not comply with repeated fetal cardiac ultrasound scans because, due to the presence of other associated fetal congenital malformations, they opted for pregnancy termination. Of the 32 prenatally verified abnormal fetal hearts (examined by both moderately experienced obstetricians and a pediatric cardiology specialist), 6 cases were interpreted as ‘minor anomaly’ (#3, 10, 28, 30, 31, and 32), while the remaining cases (n = 26) were evaluated as ‘major cardiac vitium’. CHD were considered to be ‘major anomalies’ if they required cardiac surgery during the first year of life.

Two minor anomalies that were also confirmed by the cardiologist at the first-trimester scan (#31: isolated pericardial fluid; and #32: minor inlet ventricular septal defect, VSD), which were considered as a ‘normal heart’ at the second-trimester ultrasound screening. One case of fetal septal fibrotic area detected by both the obstetrician and the cardiologist at the time of first-trimester cardiac screening was supplemented with the diagnosis of VSD during second-trimester ultrasound screening (#28). Case #30 (isolated pericardial fluid at first-trimester screening), interpreted as a ‘minor anomaly’, proved postnatally to be a major cardiac defect (complex pulmonary atresia, Table 2). 

Of the 3238 first-trimester fetal hearts that were interpreted as ‘normal’ by moderately experienced obstetricians, second-trimester fetal echocardiography was indicated due to the presence of abnormal NT, DV, or TR, that verified three further minor anomalies (VSD; case #35, 38, and 39) and one major vitium (case #36: aortic stenosis + diffuse endocardial fibroelastosis). In one case, intensive fetal cardiologic observation and follow-up were indicated due to a positive family history; however, neither first- nor second-trimester screening found any abnormality. In the third trimester, however, multiple rhabdomyomas were detected (case #37). 

### 3.2. Outcomes of Pregnancies

Of the 3270 followed fetuses, 3191 (97.6%) were born, while 79 fetuses (2.4%) were either not born or died early. Perinatal mortality was observed in 8 cases as a result of infection or for reasons that remain unclear. Major CHD was not detected in this cohort of patients. Spontaneous abortion occurred in 18 cases (0.5%) during the mid-trimester; there were no cases of major CHD in this group of patients, and first-trimester cardiac screening was negative for all of these cases. A total of 53 pregnancies (1.6%) were terminated for a genetic indication, of which 29 were found to have CHD (Table 3).

### 3.3. Postnatal Diagnoses

A total of 13 newly diagnosed minor cardiac anomalies were identified in the 3191 newborn infants (of which 10 cases of innocent cardiac murmurs and 2 cases of patent foramen ovale were excluded from the analysis as these are not considered cardiac anomalies, Table 4). In case #30, at one year of age, a pediatric cardiologic examination diagnosed a major cardiac malformation (complex pulmonary atresia, Table 3). In this particular case, the initial first-trimester cardiac scan showed a normal appearance of the four-chamber view and that of ventricular outflow tracts; however, the presence of isolated pericardial fluid was detected. Unfortunately, the pregnant patient did not attend the scheduled fetal echocardiography or the routine second and third-trimester obstetric ultrasound screenings. 

In case #32 (minor inlet VSD), second-trimester fetal echocardiography revealed that minor cardiac anomalies suspected prenatally at first-trimester screening were found to be negative, confirming a normal neonatal heart. In case #28, second-trimester fetal echocardiography augmented the diagnosis of first-trimester septal fibrotic area with VSD, and that finding was only supported partially after birth (the VSD was subsequently proved to be closed). In case #39, the initial first-trimester cardiac scan displayed a normal fetal heart, while abnormal NT was 3.3 mm, and non-invasive prenatal testing (NIPT) was negative. However, second-trimester fetal echocardiography detected a small VSD that was confirmed to be closed after birth. In the other two minor fetal cardiac anomalies diagnosed at the second-trimester fetal echocardiography, postnatal cardiac evaluation confirmed the prenatal diagnoses. 

In summary, 49 (1.49%) cardiac abnormalities were observed in the 3270 fetuses followed. Thirteen new cardiac abnormalities (28.6%) were first discovered postnatally (1 major and 13 minor defects), while 35 (71.4%) of the 49 cardiac anomalies were diagnosed prenatally (27 major and 8 minor defects).

Of the four small VSDs diagnosed prenatally, only two cases were confirmed postnatally, supporting the possibility of spontaneous intrauterine closure (healing) of minor fetal cardiac septal defects. 

Of the fetuses followed, 96.4% of major CHD (27 of 28) were diagnosed prenatally by moderately experienced obstetricians in collaboration with a pediatric cardiology specialist, and 89.2% of them (25 of 28) were already diagnosed (or at least highly suspected) at first-trimester screening and subsequent fetal echocardiography. 

In the first year of the five-year study period (2010), our objective was to visualize and store as many clear planes of the first-trimester fetal heart as possible; therefore, our reports were limited to fragments of a complete cardiac examination panel, including only a true four-chamber view plane. Of the 282 first-trimester cardiac scans conducted, only one abnormal fetal heart with an atrio-ventricular septal defect (AVSD) and one case of aortic stenosis were diagnosed in the second trimester (Table 1). During the 2011–2012 (already followed) study period, 11 major cardiac defects were identified from a significantly larger group of examinations (n = 1458), and 9 of them were picked up at the first-trimester scan. In one of the two cases where CHD was not identified (case #36, aortic atresia, left ventricle >> right ventricle, fibrosis, see Table 2), the four-chamber view (at later offline analysis the aorta appeared slightly narrower) at first-trimester scan appeared to be normal and the patient had a high risk for trisomy 21 (1:4); both were both ruled out at subsequent chorionic villous sampling (CVS). At the 18-week scan, a definitive diagnosis of fetal aortic atresia was established. The second undiagnosed cardiac vitium (detailed in the ‘results’ section) was only detected after birth (complex pulmonary atresia, case #36, see Table 2).

Of the 9 diagnosed first-trimester major cardiac defects, detailed fetal echocardiography led to the diagnosis of Down’s syndrome in two cases (case #1: AVSD and case #29: hypoplastic left heart syndrome, HLHS). In cases #2, 4, 5, and 7, fetal cardiac anomalies were identified with an NT value within the normal range and the karyotype was also normal. In one case (case #2), assessment of the situs and the four-chamber view led us to the final diagnosis of AVSD combined with heterotaxy (see Table 2). During the second half of the study (2013–2014), all major cardiac defects were identified at first-trimester screening, with the exception of one case of fetal rhabdomyoma (Table 1). 

## 4. Discussion

The clinical data of unselected consecutive pregnant women screened at our center indicate that the examined population should be considered to have an intermediate-risk profile with respect to maternal age. No statistically significant difference was observed in terms of maternal age between the entire study population and the group of pregnancies affected by major fetal CHD. In contrast, the incidence of major fetal CHD was significantly higher for the subset of women aged > 35 years.

This phenomenon highlights the increased incidence of congenital malformations, including CHD associated with advanced maternal age and chromosomal abnormalities [42,43]. The present study demonstrated that an abnormal fetal karyotype was present in 24% of cases (9/37). It is important to note that older maternal age is a significant contributing factor to fetal heart development defects. Both fetal and maternal complications increase significantly with a maternal age of 35 years during pregnancy. A maternal age of greater than 35 years has recently been associated with subclinical myocardial dysfunction [44] and several obstetrical complications (gestational diabetes, gestational hypertension, pre-eclampsia) [45,46,47].

On the other hand, a typical tendency in Hungary over the last decade is that older pregnant women with better socio-economic status prefer to undergo high-standard screening and diagnostic obstetric examinations in well-equipped specialized private obstetric clinics (with well-trained specialists) leading to a very high detection rate of various fetal pathologies. Last but not least, primary care obstetricians tend to refer their high-risk patients immediately and directly to these perinatal centers.

According to the meta-analysis, the detection rate of congenital cardiac defects increases by up to 23% [28,48]. The newly formed high-risk group of routinely screened fetuses with NT-values above the 99th percentile (cut-off > 3.5 mm) was detected. Many studies have proved that other markers for first-trimester screening for aneuploidies, such as TR and DV, are useful for screening for congenital cardiac defects. The combination of NT measurement with TR and DV blood flow assessment reaches a detection rate of 48% for major congenital cardiac defects in the first trimester [49,50].

The NT, DV, and TR measurements of our study support the data that these novel cardiac markers (NT, TR, and DV), introduced in extended first-trimester screening, can significantly narrow and refine the cohort of pregnant patients at risk for CHD beyond those at high baseline risk with classic indications. Nevertheless, the routine clinical use of NT, DV, and TR alone will not result in the detection of the vast majority of congenital cardiac defects [28,48,50,51]. To achieve this, it is essential that a structural evaluation of the fetal heart be performed in both maternal age-related and abnormal NT-, TR-, and DV-related risk pregnancy groups, as well as in the group of pregnant women at high baseline risk for CHD with classic indications.

The examinations were conducted using state-of-the-art ultrasound equipment; however, instead of high-frequency linear probes designed specifically for cardiac scanning, we used medium-frequency and resolution convex abdominal transducers with wide view-angle and greater tissue depth penetration, suitable for complete first-trimester ultrasound screening. Due to the limited maneuverability of transvaginal ultrasound probes, this approach was rarely used, mostly in cases with a retroflected uterus. Our experience supports the observation that optimal visualization is not dependent solely on body mass index (BMI) but rather on transducer-fetal heart distance, which is influenced by BMI, uterine, placental, and fetal position [28,31,32,34,52]. The extended first-trimester screening examinations were performed by three obstetricians, who had 4 to 8 years of mid-level sonographic experience in fetal diagnostics (mainly second-trimester), one-year FMF-TR/DV audit at the beginning of the study (2010), and little experience in assessing healthy and abnormal fetal heart. At the beginning of the study period, inexperience was offset by the time dedicated to the examination; some cases were analyzed offline for 3 to 4 h. Later on, a highly-qualified sonographer joined our team and her excellent overall performance in the pre-screening allowed the obstetricians to focus on the evaluation of pathologic findings and, subsequently, the total examination time was reduced to 20–30 min. A pre-screening ultrasound by a sonographer should always be followed by post-screening performed by an obstetrician preferably using check-lists to ensure continuous quality control.

Previously, our pediatric cardiologist was used to performing echos on fetuses over 16 weeks, but thanks to her openness, she became more and more comfortable with first-trimester ultrasounds. By the end of the study period, fetal hearts interpreted as ‘abnormal’ by moderately experienced obstetricians were rescanned by the pediatric cardiologist and pregnant mothers received a detailed fetal cardiologic second opinion within 24 h, significantly reducing their psychological distress.

Case #36 supports the novel findings that the progression of CHD may be intense, especially up to the 20th week of pregnancy.

In the initial period of the learning curve (2010–2012), the typical and common features of the nine cases of CHD detected in the first trimester were an abnormal four-chamber view and failure to demonstrate the involvement of the great vessels in the cardiac defect. (A limitation of each study, including ours, which addresses the prenatal sonographic assessment of abnormal hearts, is that subsequent histopathological evaluation of the first-trimester fetal heart is not feasible in the majority of cases). Although the assessment of outflow tracts did not contribute to the diagnosis of CHD, we believe that it was nevertheless an invaluable experience. The long timeframe available for extended first-trimester screening resulted in significantly enhanced proficiency in delicate transducer movements and empirical learning.

Table 1 shows that during the second half (2013–2014) of our study, first-trimester abnormal great-vessel diagnoses were also observed (Tetralogy of Fallot, Transposition of the Great Arteries).

## 5. Conclusions

According to our research, if appropriate equipment, quality control, and motivation are in place, the effectiveness of first-trimester fetal cardiovascular ultrasound screening by moderately experienced obstetricians in an unselected (so-called “routine”) pregnant population may reach 90% in terms of major congenital heart defects.

Our data demonstrate that early fetal echocardiography not only provides reassurance to the majority of pregnant women with regard to the absence of cardiac anomalies, as the most common congenital birth defect, but also significantly contributes to the early diagnosis of chromosomal abnormalities. The majority of cases in which fetal cardiac anomalies were detected are usually so complex and severe that they allow couples to opt for an early termination of their pregnancies and thus decrease maternal general medical risks and the psychological burden of second-trimester elective termination.

The use of first-trimester cardiac markers (NT, TR, and DV) may draw our attention to serious complex structural heart defects and may also help in the detection of minor and major fetal cardiac anomalies that are only visualized at second-trimester screening. Subsequently, it may provide an opportunity to improve neonatal morbidity and mortality as a result of prior organization of intensive perinatal cardiac care.

Second-opinion fetal echocardiography, performed by pediatric cardiology specialists to re-assess suspected cardiac anomalies by moderately experienced obstetricians, is crucial as correction and/or augmentation of established diagnoses will help genetic counselors and couples to better understand the severity, complexity, and prognosis of the condition.

The immediate availability of a pediatric cardiologist can significantly reduce the psychological stress that parents experience as a result of the diagnostic uncertainty of the obstetricians.

In our study, we presented a 5-year learning curve, at the conclusion of which we had performed a high case number of nearly 4800 first-trimester fetal cardiac scans, identified over 40 fetal cardiac anomalies, and significantly enhanced proficiency among physicians in visualizing and assessing both four-chamber view- and outflow tract-planes, as well as large vessel relations of the 11 to 13-week fetal heart.

It may be reasonably assumed that an experienced obstetrician-sonographer performs at least 1300 first-trimester aneuploidy and cardiovascular screens annually. Consequently, in a country like Hungary, only 10–15% of moderately and highly experienced obstetrician-sonographers would require specialized training to enable them to perform detailed early fetal echocardiography, thereby ensuring that every pregnant Hungarian woman has access to this service. Provided that a structured high-quality training program is made available, this could be achieved in a shorter time span than our learning curve, and thus first-trimester fetal echocardiography might become part of routine first-trimester screening.

## Figures and Tables

**Table 1:** Prenatally observed abnormal hearts during the learning curve.

	2010	2011	2012	2012	2014	Total
# of 1st-trim. examinations	228	586	872	1228	1855	4769
# of abnormal hearts (1st-trim. Dx)	2 (1)	8 (7)	6 (5)	15 (14)	11 (9)	42 (36)
HLHS		2		1	1	4
AVSD	1	1	1	1	3	7
AVSD + HRH		1				1
AVSD + HLH			1		1	2
AVSD + Heterotaxia		1				1
VSD		1	2	3	1	7
VSD + LV < RV				1		1
ASD + LV < RV			1			1
LV > RV					1	1
LV < RV					1	1
HLH + VSD				1		1
HRH + VSD		1				1
Aortic stenosis + HLH + AVSD				1		1
Aortic atresia	1	1				2
Pulmonary valve regurgitation					1	1
Pulmonary atresia + HRH				1		1
Septal fibrosis + VSD				1		1
Ventricular fibro-elastosis				1		1
Rhabdomyoma					1	1
TGA					1	1
Tetralogy of Fallot				1		1
Isolated pericardial fluid			1	1		2
CHD—non evaluated				2		2

Abbreviations: #, number; Dx, diagnosis; HLHS, hypoplastic left heart syndrome; AVSD, atrio-ventricular septal defect; HRH, hypoplastic right heart; HLH, hypoplastic left heart (LV << RH); VSD, ventricular septal defect; LV, left ventricle; RV, right ventricle; ASD, atrial septal defect; TGA, transposition of great arteries; CHD, congenital heart defect.

**Table 2:** Abnormal cardiac findings during the following period.

	1st-Trim. OB. Dx	1st-Trim. Card. Dx	NT	TR	Abn. DV	20-Week OB + Card.	Assoc. Malformations	Karyotype	Pregnancy Outcome
1	AVSD	idem	2.3	+	+	-		47XX, +21	Termination
2	AVSD, HeterotaxiaDextrocardia	idem	1.7	not done	+	-	-	-	Termination
3	VSD	idem	7.9	+	+	-	-	-	Termination
4	AVSD + HRH	id. + fibro-elast	2.5	-	+	-	SUA	46XY	Termination
5	AVSD + HLHS	idem	1.9	-	-	-	-	46XX, 16 polymorph.	Termination
6	LV < RV, ASD	Not examined	5.1	-	-	-	Omphalocele paklatoschisis	47XX, +13	Termination
7	Tricu. atr, HRH, VSD	idem	1.6	Flow:-	-	-	-	46XX (abortum)	Termination
8	AVSD	idem	5	+	_	-	Oligohydramnios	-	Termination
9	HLHS	idem	6	+	-	-	Holoprosencephalia Polydactylia	47XY, +13	Termination
10	VSD (subaortic)	idem	N/A	not done	not done	-	Hydronephrosis Camptodactylia SUA oligohydramnion	69XXX	Termination
11	VSD, fibro-leastosis	idem	2.1	-	-	-	-	-	Termination
12	PA atr., HRH	idem	3.9	-	-	-	-	-	Termination
13	RV fibro-elastosis	idem	5.5	not done	not done	-	-	-	Termination
14	HLHS, VSD	HLHS, AVSD Aorta stenosis	1.5	+	not done	-	-	-	Termination
15	Left rot., Abn. GA	Not examined	6	not done	+	-	cervical cyst, short bones	PCR negative Cytogenetics cannot be performed:	Termination
16	HLHS	idem	2.4	-	+	-	Alob. holoprosencephaly Palatoschisis SUA	-	Termination
17	HLHS, VSD,	idem	7	-	not done	-	Holoprosencephaly Encephalocele Omphalocele	-	Termination
18	Abn. 4-CV + outfl. tr.	Not examined	8	not done	+	-	-	-	Termination
19	LV < RV, VSD	idem	7.3	-	-	-	Cleft lip and palate	47XX, +13	Termination
20	Tetralogy of Fallot	idem	4.5	.	+	-	-	-	Termination
21	LV < RV, AVSD	only AVSD	3.6	-	+	-	-	47XX, +21	Termination
22	AVSD	idem	4.7	+	+	-	Clubfoot, short bones	-	Termination
23	LV < RV,	idem	9	not done	+	-	Strawberry shape skull	-	Termination
24	RV > LV, P. valve reg.	P.valve reg.	1.8	+	+	-	-	47XX, +17 Chr 16 polymorph.	Termination
25	AVSD	idem	5.1	+	+	-	Short bones	-	Termination
26	AVSD	idem	6.5	+	+	-	-	-	Termination
27	HLHS	idem	1.2	not done	+	-	Omphalocele Cheilo-palatoschisis	-	Termination
28	Septal fibrotic area, Rhabdomyoma	idem	1.6	-	-	idem + VSD; Swiss cheese VSD	-	46XX	Born, idem, closed VSD No VSD Ventricular septum in upper third of echo-dense terime
29	HLHS	idem	3.1	+	+	-	-	47XX, +21	Termination
30	Isol. pericard. fluid	idem	2.1	-	-	hasn’t come back	-	-	Born, complex P. atresia 1 year old with com-plex pulmonary atresia
31	Isol. pericard. fluid	idem	1.8	-	-	normal heart	-	-	Born, healthy
32	Large VSD	Min. inlet VSD	2.8	-	-	normal heart	-	46XX	Born, healthy
33	RV < LV	normal heart	4.8	+	-	normal heart		46XX, NT panel negative	Born, healthy
34	PA < Ao	normal heart	2.1	-	-	normal heart		-	Born, healthy
35	normal heart	-	2.4	-	-	VSD (subaortic)	Cleft lip	46XY	Born, VSD closed
36	normal heart	-	2.4	-	+	Ao. atr, LV > RV, fibr endocardialis fibrosis		46XY	Termination
37	normal heart	-	2	-	-	3-trim. Rhabdomyoma; Multiplex rhabdomyoma	-	-	Born, Dx proved
38	normal heart	-	1.9	-	+	VSD (inlet)		46XY	Born, Dx proved
39	normal heart	-	3.3	-	-	small VSD	-	NIPT: negative	Born, cl. lip, VSD closed

Abbreviations: HLHS, hypoplastic left heart syndrome; AVSD, atrio-ventricular septal defect; HRH, hypoplastic right heart; HLH, hypoplastic left heart (LV << RH); VSD, ventricular septal defect; LV, left ventricle; RV, right ventricle; ASD, atrial septal defect; TGA, transposition of great arteries; CHD, congenital heart defect; Dx, diagnosis; OB, medium-experienced obstetrician; Card, pediatric cardiologist specialist; NT, nuchal translucency; TR, tricuspidal regurgitation, DV, ductus venosus; Abn, abnormal; Assoc, associated; Tricu atr, tricuspidal atresia; PA, pulmonary artery; Rot, rotation; GA, great arteries; 4-CV, four-chamber view; Outfl. tr., Outflow tracts; P. valve reg., Pulmonary valve regurgitation; Pericardial, pericardial; Ao, Aorta; idem (id), identical; N/A, non-available; SUA, single umbilical artery; Alob, alobar; cl. lip, cleft lip.

**Table 3:** Outcome of pregnancy in the follow-up period.

Number of Pregnancies Followed	n = 3270
Live births	3191 (97.6%)
Pregnancy losses	79 (2.4%)
Perinatal deaths (intrauterine demise + neonatal death)	8 (0.2%)

**Table 4:** Postnatally recognized cardiac findings.

Type of Cardiac Anomaly	Number of Fetuses
Ventricular septal defect (2–5 mm)	8
Atrial septal defect II	3
Aortic valve stenosis min. grade	1
Bicuspidal aortic valve + PFO	1
Complex pulmonary atresia	1
TOTAL	14
(PFO + innocent cardiac murmurs)	(2 + 10)

Abbreviation: PFO: patent foramen ovale.

## Data Availability

The published article contains all generated and analyzed data from this series.
